# Arterial embolization for massive bleeding from a locally advanced breast tumor

**DOI:** 10.1016/j.radcr.2024.07.050

**Published:** 2024-08-13

**Authors:** Thomas Le Tat, Raphaël Jost, Viseth Kuoch, Robert-Yves Carlier, Mostafa El Hajjam, Jeffery Zhou

**Affiliations:** a Department of Interventional Radiology, Centre Hospitalier Sud Francilien, 40 Avenue Serge Dassault, Corbeil-Essonnes 91100, France; b Department of Radiology, Hôpital National d'Instruction des Armées Percy, 2 Rue Lieutenant Raoul Batany, Clamart 92140, France; c Department of Radiology, Hôpital Ambroise Paré (AP-HP), 9 Av. Charles de Gaulle, Boulogne-Billancourt 92100, France

**Keywords:** Arterial embolization, Breast Cancer, Interventional radiology, Bleeding

## Abstract

Advances in breast cancer treatment have markedly reduced the incidence of massive bleeding, yet severe hemorrhage remains a critical issue in locally advanced or metastatic cases. Traditional management strategies often prove inadequate for significant bleeding, highlighting the need for alternative interventions. We detail the management of a 64-year-old patient with a neglected locally advanced breast tumor, leading to life-threatening hemorrhage. Conventional bleeding control measures failed, necessitating microsphere embolization. Effective hemostasis was achieved without adverse events or recurrence of bleeding, allowing for the initiation of chemotherapy. This case underscores the rarity yet potential severity of hemorrhage in breast cancer, challenging conventional management. Embolization, typically reserved for other hemorrhagic conditions, is appearing as a viable alternative for breast cancer-related hemorrhage, particularly in large tumors where surgery is impractical. Further research is necessary to establish its role in managing minor bleeding.

## Introduction

Advancements in breast cancer therapy have significantly reduced the incidence of massive bleeding. However, in the context of locally advanced or metastatic breast cancer, bleeding can progress to life-threatening severity. Topical treatments and radiotherapy, while effective for minor oozing, are inadequate for controlling severe hemorrhage. Surgical intervention, a potential remedy for such bleeding, is associated with considerable morbidity in advanced cancer stages. Hemostatic embolization, although infrequently mentioned for this indication, emerges as an outstanding alternative.

## Case report

We report the case of a 64-year-old patient with a history of arterial hypertension presenting with a neglected mass in the left breast, characterized by skin ulceration and notable swelling. Biopsy revealed a 19 cm pleomorphic lobular carcinoma that was estrogen receptor positive (100%), progesterone receptor positive (30%), Ki67 (60%), and HER2 1+. Both TAP and PET scans indicated no evidence of metastasis.

**Fig. 1:**
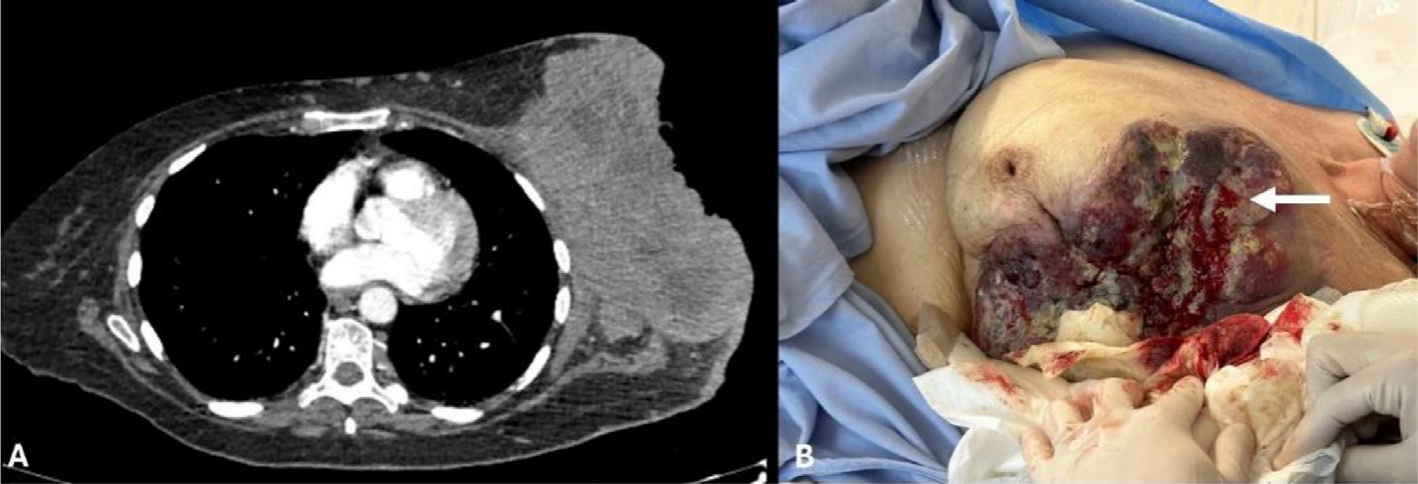
(A) CT scan showing the voluminous left breast mass. (B) Intraprocedural view showing the persistence of moderate bleeding (arrow) after embolization of the internal thoracic artery. This bleeding stopped after embolization of the axillary artery branches.

One month following the biopsy, and prior to the initiation of anticancer therapy, the patient presented with severe hemorrhage from the tumor, necessitating emergency intervention (Fig.1). Initial management with a pressure dressing temporarily halted the bleeding, which resumed intensely within 24 hours, resulting in significant anemia (hemoglobin level: 7.5 g/dL), tachycardia (blood pressure: 110/70 mmHg; heart rate: 110 bpm) and precluding surgical intervention due to the associated high risk.

**Fig. 2:**
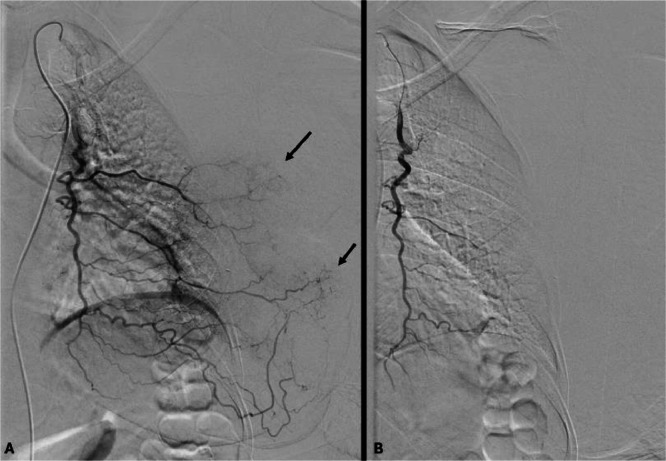
(A) Angiography of the internal thoracic artery showing tumor blush (arrows) without active bleeding. (B) Control after embolization with 500-700 µm embospheres®, showing disappearance of tumor blush.

**Fig. 3:**
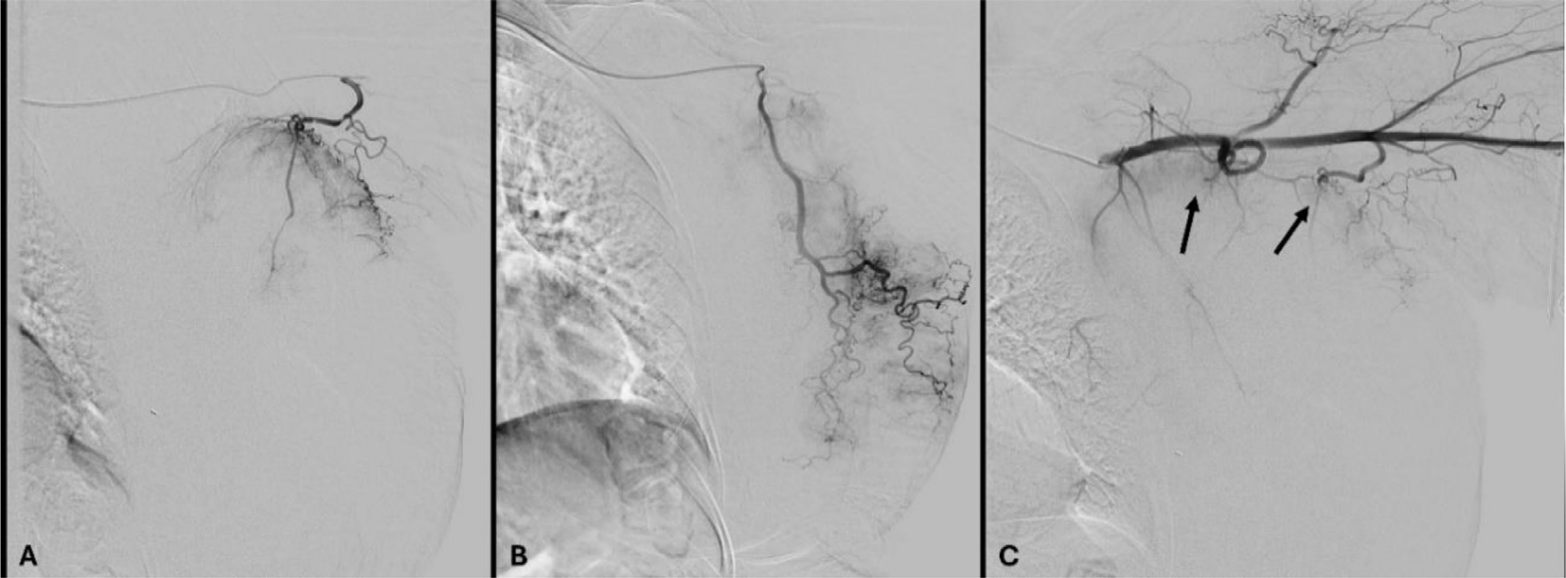
(A and B) Selective angiography of axillary artery branches showing tumor blush without active bleeding (C) Control after embolization of these 2 arteries with 500-700 µm embospheres®, showing a slight persistent tumor blush (arrows). As there was no longer any clinical bleeding, it was decided to stop the procedure to avoid excessive ischemia with a risk of skin necrosis. If the bleeding had persisted, it might have been necessary to embolize intercostal arteries.

The hemostatic procedure was performed under local anesthesia in an interventional radiology suite. Utilizing ultrasound guidance, the right common femoral artery was punctured, and the left internal thoracic artery (Fig. 2) along with 2 branches of the axillary artery (Fig. 3) were catheterized using a 5 French internal mammary catheter (Cordis, Santa Clara, USA). Initial angiography demonstrated substantial tumor blush absent of active hemorrhage. Through microcatheterization employing a Progreat 2.7 Fr microcatheter (Terumo, Tokyo, Japan), these arteries were embolized using 3.5 mL of Embosphere® 500-700 µm (Meritmedical, South Jordan, USA).

Bleeding was effectively controlled after embolization of these arteries, negating the need for intercostal artery investigation. The patient was subsequently transferred to her designated oncology clinic 5 days postprocedure to initiate chemotherapy treatment. At follow-up 3 months later, there was no evidence of bleeding recurrence.

## Discussion

Breast cancers typically are less prone to hemorrhaging compared to renal or other tumor types. Minor bleeding often associated with skin lesions can usually be managed with topical treatments and diligent skin care [1]. Furthermore, radiotherapy has demonstrated efficacy in controlling small, chronic bleeding [2].

However, significant hemorrhage may occur, albeit rarely, if the tumor's vascularizing small vessels are compromised. While mastectomy can offer a solution for bleeding control, it may not always be feasible, especially in cases of large tumors like that of our patient. In such scenarios, embolization presents itself as an optimal alternative, offering effectiveness and tolerability without the need for general anesthesia.

Given the rarity of massive bleeding secondary to breast tumors, embolizations for this purpose are not commonly reported [[3], [4], [5], [6], [7]]. The case detailed here explores this procedure further. A notable risk of embolization is the unintended embolization of nontarget areas. Specifically, for breast cancer, where blood supply primarily originates from subclavian and axillary artery branches, the most critical potential complications include embolization affecting the upper limb and the vertebrobasilar territory. Similar caution is warranted for possible medullary artery embolization when the source is an intercostal artery, akin to precautions during bronchial artery embolization. The literature reports no such complications in breast cancer embolizations for hemostatic or anticancer objectives [[3], [4], [5], [6], [7], [8], [9], [10], [11]]. Localized advanced tumors often invading the skin may result in skin ischemia postembolization, potentially leading to significant pain, which in our case was managed with 2 mg of intraoperative morphine.

Various embolization agents are available; however, microspheres are usually preferred for ``definitive'' tumor bleeding management over porcine gelatin, and over coils or plugs that could hinder future embolizations [[12], [13], [14]]. While liquid agents like Glubran 2® (GEM, Viareggio, Italy) and Onyx® (Medtronic, Dublin, Ireland) are options, their higher costs and the complexity of distal microcatheterization, particularly in elderly, atherosclerotic patients, limit their use. Given the relative ease of catheterizing internal and lateral thoracic arteries—key breast blood suppliers—embolization could be considered even for less severe bleeding that impacts quality of life or in cases necessitating anticoagulant therapy.

## Conclusion

Embolization offers an effective and safe intervention for managing significant hemorrhage associated with breast tumors, especially when surgical options are limited due to tumor size. Further research is warranted to evaluate the efficacy of embolization in managing minor bleeding episodes.

## Patient consent

Written, informed consent was obtained from the patient for publication of this case.
